# Surface EMG-based Sketching Recognition Using Two Analysis Windows and Gene Expression Programming

**DOI:** 10.3389/fnins.2016.00445

**Published:** 2016-10-14

**Authors:** Zhongliang Yang, Yumiao Chen

**Affiliations:** ^1^College of Mechanical Engineering, Donghua UniversityShanghai, China; ^2^Fashion and Art Design Institute, Donghua UniversityShanghai, China

**Keywords:** sketching, surface electromyography, gene expression programming, muscle-computer interface, pattern recognition

## Abstract

Sketching is one of the most important processes in the conceptual stage of design. Previous studies have relied largely on the analyses of sketching process and outcomes; whereas surface electromyographic (sEMG) signals associated with sketching have received little attention. In this study, we propose a method in which 11 basic one-stroke sketching shapes are identified from the sEMG signals generated by the forearm and upper arm muscles from 4 subjects. Time domain features such as integrated electromyography, root mean square and mean absolute value were extracted with analysis windows of two length conditions for pattern recognition. After reducing data dimensionality using principal component analysis, the shapes were classified using Gene Expression Programming (GEP). The performance of the GEP classifier was compared to the Back Propagation neural network (BPNN) and the Elman neural network (ENN). Feature extraction with the short analysis window (250 ms with a 250 ms increment) improved the recognition rate by around 6.4% averagely compared with the long analysis window (2500 ms with a 2500 ms increment). The average recognition rate for the eleven basic one-stroke sketching patterns achieved by the GEP classifier was 96.26% in the training set and 95.62% in the test set, which was superior to the performance of the BPNN and ENN classifiers. The results show that the GEP classifier is able to perform well with either length of the analysis window. Thus, the proposed GEP model show promise for recognizing sketching based on sEMG signals.

## 1. Introduction

Conceptual design is one of the earliest stages of product development and is responsible for defining key aspects of the final product (Briede-Westermeyer et al., 2014). Designers commonly use freehand sketching to express conceptual design, which is a reflection-in-action process that occurs during design (Schön, 1983). As a natural and efficient method to communicate ideas (Van der Lugt, 2005), the potential advantages of sketching have been widely recognized and exploited in many fields (Pu and Gur, 2009).

Sketching is a visual language, and as with any language, some elements go together to form more structured forms of communication. However, there is no definitive set of basic shapes. The most common are: line/curve/arc, rectangle/square/diamond, circle/ellipse, arrowhead and triangle (Schmieder et al., 2009). However, novice designers are trained to precisely and quickly draw four basic shapes with one stroke: freehand straight lines, ellipses, circles and smooth curves (Olofsson and Sjölén, 2007; Robertson and Bertling, 2013). Design sketches can be sketched with an arbitrary number of these four kinds of basic one-stroke shape.

Pen and touch medium tablets, interactive pen displays, touch screens, tablet computers, and mouse have typically been used to record and transmit sketching messages to computers. Many of the most robust sketch recognition systems today focus on gesture or isolated symbol recognition (Field et al., 2010). Hidden Markov models (Anderson et al., 2004; Cao and Balakrishnan, 2005; Sezgin and Davis, 2005), neural networks (Pittman, 1991), feature-based statistical classifiers (Rubine, 1991; Cho, 2006), dynamic programming (Myers and Rabiner, 1981; Tappert, 1982), *ad-hoc* heuristic recognizers (Wilson and Shafer, 2003; Notowidigdo and Miller, 2004), proportional shape matching (Kristensson and Zhai, 2004) and $1 recognizer (Wobbrock et al., 2007) have been widely used for gesture recognition. Despite substantial progress in this area, recognizing sketching remains a difficult problem since sketches are informal, ambiguous, and implicit (Li, 2003).

A recent trend is toward more accessible and natural interfaces. Thus, Human Computer Interaction (HCI) has drawn attention to the voice, hand gesture and posture as important perceptual interfaces in addition to the traditional computer peripherals or touch screens (Linderman et al., 2009; Chen et al., 2015). An interface that converts human bioelectric activity to external devices, known as bioelectric interface (Linderman et al., 2009), especially muscle-computer interface (Chowdhury et al., 2013), has become a new research focus in the field of HCI. Sketching and its key role in concept design are identified (Schütze et al., 2003; Tovey et al., 2003) and sketch-based interfaces can achieve more natural HCI (Kara and Stahovich, 2005), which has led to computer-aided sketching (CAS) systems (Sun et al., 2014) and sketch-based interfaces for modeling in computer-aided design (CAD) systems (Olsen et al., 2009). Although much progress has been made, sketching has received little attention from the designers of bioelectric interfaces due to perceived technical limitations (Linderman et al., 2009), the paucity of models (McKeague, 2005), and intra-class variations and inter-class ambiguities of sketches (Li et al., 2015).

Surface electromyographic (sEMG) signals have been used to control computers (Wheeler and Jorgensen, 2003), prosthesis (Farina et al., 2014; Jiang et al., 2014), robots (Kiguchi and Hayashi, 2012) and wheelchair (Andreasen and Gabbert, 2006). Researchers also have used EMG as new interfaces (Ahsan et al., 2009; Chowdhury et al., 2013) for recognition of hand gestures (Chen et al., 2007; Fougner et al., 2014), sign languages (Li et al., 2010), body languages (Chen et al., 2014) and facial expressions (Chen et al., 2015). However, the sEMG, which reflects to some extent the underlying neuromuscular activity (Jian, 2000), has also been found useful for the recognition of handwriting (Lansari et al., 2003; Linderman et al., 2009; Huang et al., 2010; Chihi et al., 2012). For instance, Linderman et al. (2009) showed a method in which EMG signals generated by hand and forearm muscles during handwriting activity are reliably translated into both algorithm-generated handwriting traces and font characters using decoding algorithms. Huang (2013) proposed to use the dynamic time warping algorithm for the overall recognition of handwriting signals of the EMG. The fact that brain, hand, and eye actions are tightly connected in the sketching process (Goel, 1995; Taura et al., 2012; Sun et al., 2014) suggests that bioelectric interfaces potentially could extract normal sketching patterns directly from hand and arm EMG signals. However, this question has rarely been explored so far.

Two things are needed to improve the classification accuracy of subtle sketching movements: feature extraction and recognition model construction (Englehart and Hudgins, 2003; Nielsen et al., 2011). Selecting a proper length of analysis window and increment for feature extraction can improve classification accuracy (Smith et al., 2011; Earley et al., 2016). Any algorithm that enables recognizing the sketching patterns from the features of sEMG signals should be adopted (Nielsen et al., 2011). It is expected that advanced algorithms would satisfy the following requirements (Farina et al., 2014; Hahne et al., 2014): little user training, high computational efficiency and also performing well with few electrodes. Those aspects are addressed in the present study by applying a robust variant of genetic programming, namely Gene Expression Programming (GEP).

This study proposes an sEMG-based method for the recognition of 11 basic one-stroke shapes from sketching in conceptual design. More specifically, our distinctive contributions are: (1) A new experiment protocol will be established to record the sEMG signals from 4 forearm and 2 upper arm muscles of 4 participants who will be instructed to trace and cover each printed one-stroke shape on sketching templates; (2) we will extract 180 time domain indices of the sEMG signals with a short analysis window and 18 indices with a long analysis window. (3) After reducing dimensions with principal component analysis (PCA), GEP, Back Propagation neural networks (BPNN) and Elman neural networks (ENN) will be used to construct recognition models respectively for comparison.

## 2. Materials and methods

### 2.1. Participants

This study was approved by the Ethics Committee of Donghua University. Four healthy graduate students majoring in industrial design (2 males and 2 females; mean ± SD, age = 23.5 ± 0.58 years, height = 170.75 ± 8.61 cm, and weight = 65 ± 11.01 kg) volunteered to participate in the experiment. All participants had a medical examination to exclude upper limb musculoskeletal and nervous diseases, and they are right-handed. Before the experiment, they promised not to do any forearm or hand strenuous exercise.

### 2.2. Equipment and materials

#### 2.2.1. Electromyographer

The sEMG signals were collected, amplified and transmitted using a 12-channel digital EMG system (ZJE-II, ZJE Studio Ltd., China). It has an amplifier gain of 1000, conditioned with a digital band-pass filter between 10 and 500 Hz with a notch filter implemented to remove the 50 Hz line interference. The sEMG signals underwent a 14 bit analog to digital conversion at a sampling frequency of 1000 Hz.

The single disposable Ag/AgCl strip electrodes (5 cm in length and 3.5 cm in width), filled with conductive electrode paste (Jun Kang Medical Supplies Ltd., China) were used to measure sEMG activity. The electrodes can be snapped onto the EMG cable that connects it to the EMG amplifier.

#### 2.2.2. Tested shapes and sketching materials

Table 1 shows the tested one-stroke shapes that are variations of the four basic one-stroke shapes. We selected in total 11 one-stroke shapes: horizontal, vertical, 45° and 135° straight lines, horizontal 20° and 40° ellipses, vertical 20° and 40° ellipses, perfect circles, S curves, and arcs. The selection of these tested shapes was based on the fact that these were short, well-known and frequently used shapes among the subjects in product design. The degree of an ellipse is the measure of the angle of the line of sight into the surface of the ellipse (Robertson and Bertling, 2013).

**Table 1:** **Tested one-stroke shapes**.

**Straight lines**	**Ellipses**	**Circles**	**Smooth curves**
Horizontal straight line	Horizontal 20° ellipse	Perfect circle	S curve
Vertical straight line	Horizontal 40° ellipse		Arc
45° straight line	Vertical 20° ellipse		
135° straight line	Vertical 40° ellipse		

**Figure 1:**
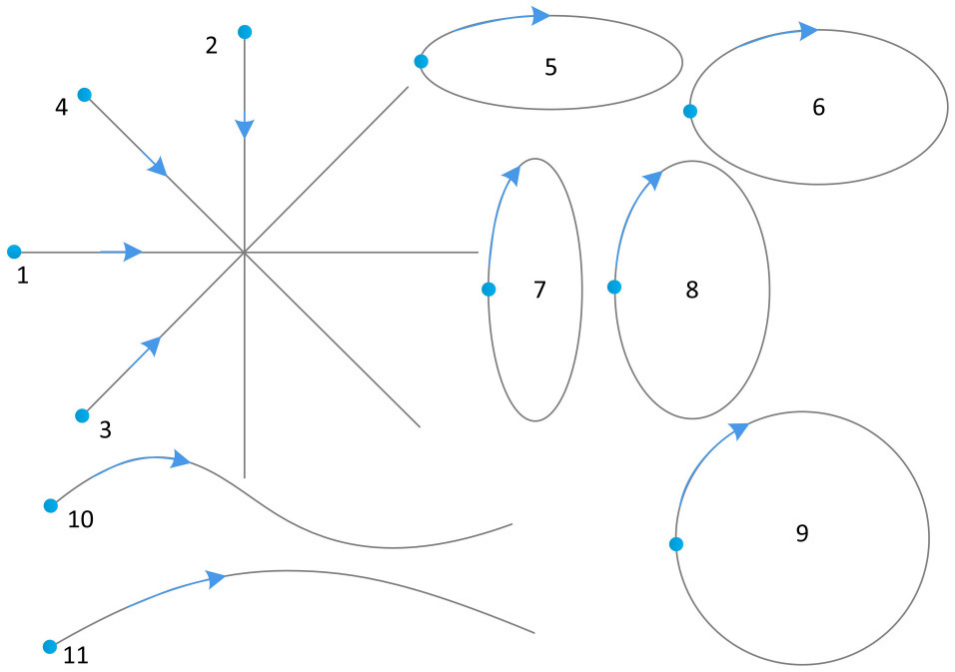
**One example of the sketching templates (dots represent starting points, arrows represent directions)**.

We printed these 11 one-stroke shapes into a piece of A4 paper with shallow color as a sketching template. We prepared ten patterns of template paper consisting of 11 shapes each. The order of the 11 shapes on the list of sketching template paper was randomized, with a different random order for each pattern. Subjects were required to draw on the paper to trace and cover each printed shape. Figure 1 shows an example of the sketching templates.

A standard mesh computer chair with a 40 cm seat height and rectangular wooden desktop (length: 100 cm, width: 60 cm, height: 75 cm) was used to replicate the conditions that are found in a regular work room. Subjects sketched on a sketching template, using a blue ink ballpoint pen, model BIC Cristal, with a hexagonal barrel, a medium point of 1.0 mm and line width of 0.4 mm.

#### 2.2.3. Electrode placement

After the location of muscles through palpation during voluntary contraction, the skin of each subject was shaved where necessary and then carefully cleaned with alcohol and electrolyte gel to reduce contact impedance and improve the electrical and mechanical contact of the electrodes (de Almeida et al., 2013). The electrodes were fixed to the skin with hypoallergenic tape to reduce movement artifacts and minimize interference in the sketching performance.

**Figure 2:**
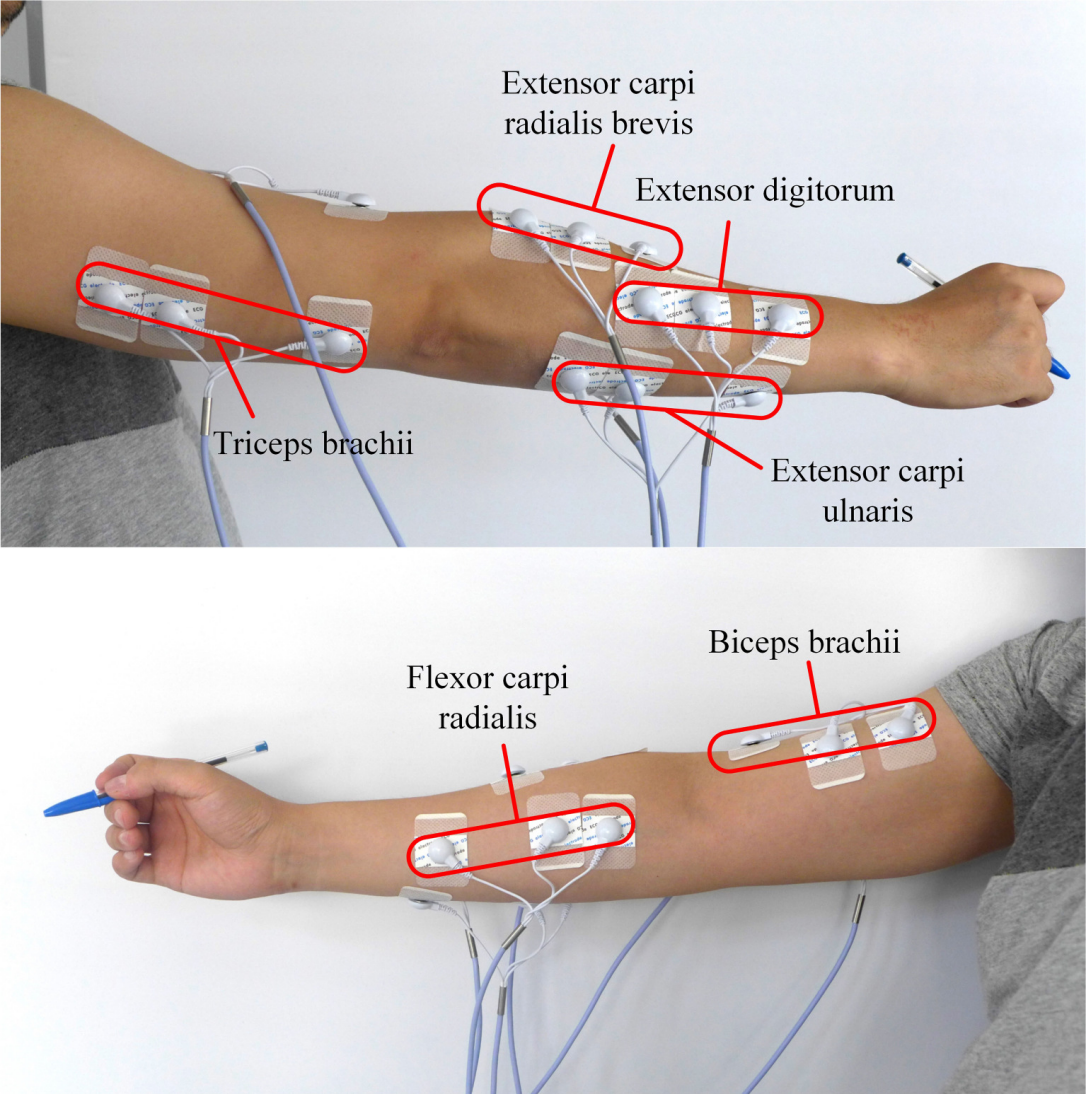
**Electrode placement over the forearm and upper arm muscles**.

Previous research (Lansari et al., 2003; Huang et al., 2010; Chihi et al., 2012) always recorded surface EMGs from hand muscles for handwriting recognition. However, according to Robertson and Bertling (2013) and our experience, designers should move the whole arm to sketch. Researchers have also proposed that the increasing number of tested muscles has a positive effect on improving the accuracy of recognition (Linderman et al., 2009). Since sketching involves the finger, wrist, and whole arm movements, sEMG signals can be recorded from intrinsic hand, forearm and upper arm muscles. However, the pen is prone to touching sEMG electrodes attached on hand to arise electrode shift, which will make noises during the collection of EMG signals, and reduce the recognition accuracy. Besides, placing electrodes on hand skin is not accessible and natural for practical use. Therefore, only EMG activity over the forearm and upper arm muscles was measured for feature extraction. Thus, four forearm muscles: flexor carpi radialis (FCR), extensor digitorum (ED), extensor carpi ulnaris (ECU), extensor carpi radialis brevis (ECRB) and two upper arm muscles: triceps brachii (TB) and biceps brachii (BB) were selected for their major role in stabilization and movement of the upper limb during fine dexterity activities, such as handwriting and sketching (Linderman et al., 2009; de Almeida et al., 2013). The locations of the surface EMG electrodes are shown in Figure 2.

### 2.3. Experimental protocol

The experiments were carried out for 10 days in October in 2015. They were performed twice a day, one from 09:00 to 12:00 for women and another from 14:00 to 17:00 for men. The experiment was divided into three stages: a welcome stage, a preparation stage, and a task stage. During a welcome stage, the procedures and the equipment used for the experiment were introduced to the participants. All participants were required to sign a consent form with a detailed description of the experiment and complete a background questionnaire about personal information such as height and weight.

During the preparation stage, the task instructions were read to the participants, and they were given a brief tutorial on how to complete the task. The objective was to reproduce as accurately as possible the natural conditions of designers in a workroom environment. Participants were instructed to practice the task prior to the data acquisition as many times as necessary to feel secure about their performance and were informed that they would be given a sketching template paper. Then they were asked to sketch on the paper to trace and cover each printed one-stroke shape, using their trained pen grasp posture and sketching skill in one stroke.

For the task stage, we prepared 100 sheets of sketching template paper consisting 11 one-stroke shapes for each participant. By a trial, we define a recording epoch during which a subject sketches a single one-stroke shape. Each shape was successively sketched 100 times. The obtained data were used as the training and test set. Therefore, each subject sketched 1100 shapes (i.e., performed 1100 trials) during the whole experiment. Since we sought to recognize basic one-stroke shapes, the subjects were asked to make pauses between the one-stroke shapes. The trials were paced by the timer software of mobile phone which played a beep sound at the beginning of each trial. The duration of each trial was 3 s of which 1–2.5 s corresponded to each shape sketching.

**Figure 3:**
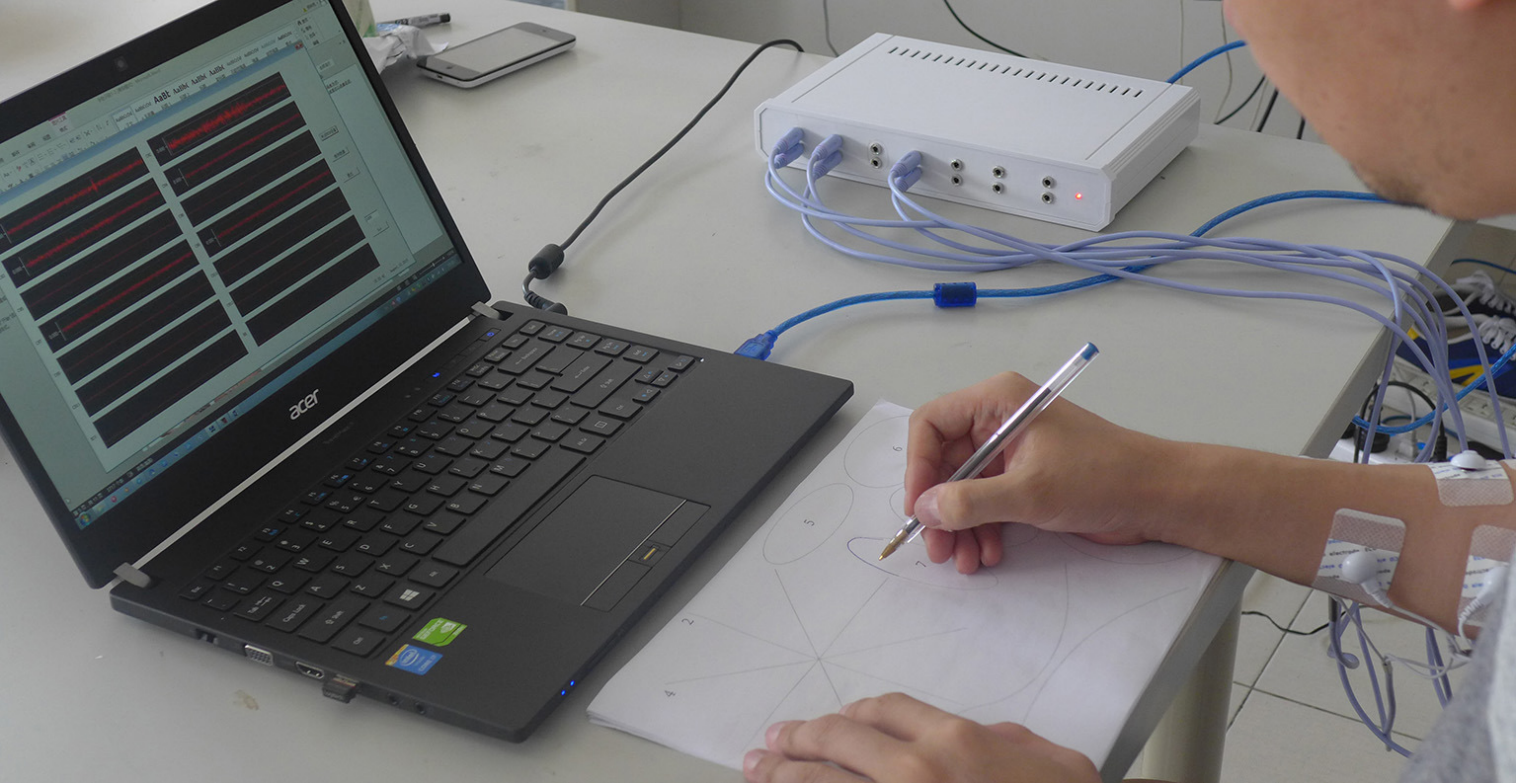
**A photograph of an experimental session**.

At the beginning of the task stage, electrodes were attached to the participants' skin and connected to the EMG system. The locations were marked on skin with a marking pen to ensure the same locations during every session. In each session, new electrodes were attached again on the pen marks. Next, a sheet of sketching template paper was presented to the participant. Each repetition of the task was initiated with the subject holding the pen with their usual grasp pattern at a desk in front of a new sketching template. The subject was instructed to remain in this position until the maximal relaxation point, and simultaneous visualization of the EMG signal was registered, as shown in Figure 3. At this point, the beginning of the task was requested through a beep sound. EMGs of 6 muscles were simultaneously recorded. Each subject was instructed to sketch on the sketching template to trace and cover printed stimuli. To avoid muscle fatigue, subjects would rest for 5 min after each set of tested shapes (i.e., one sheet of sketching template paper) and the collection of EMG signals was stopped, but the electrodes were not removed until the subject finished ten sets of tested shapes.

The design of the experiment was a One-stroke shape (11) × Repetition (10) × Day (10) within-subjects design, amounting to 1100 trials per participant. Participation in the experiment took approximately 600 min.

### 2.4. Feature extraction

Among the various ways for sEMG feature extraction, the average Electromyography (aEMG), Root Mean Square (RMS) and Mean Absolute Value (MAV) are able to respond to the changes of signal amplitude characteristics (Jian, 2000; Ren et al., 2004; Poosapadi Arjunan and Kumar, 2010; Geethanjali and Ray, 2011) and are widely used in the sEMG pattern recognition. Therefore, this paper combined these three time domain characteristics as features. These features were extracted separately from 6 channels of sEMG signals during the sketching of each shape. The aEMG, RMS and MAV are computed as follows:

(1) $$\begin{array}{lll} aEMG & = & \frac{1}{N}\overset{N}{\underset{i=1}{\sum }}v_{i} \end{array}$$

(2) $$\begin{array}{lll} RMS & = & \sqrt{\frac{1}{N}\overset{N}{\underset{i=1}{\sum }}v_{i}^{2}} \end{array}$$

(3) $$\begin{array}{lll} MAV & = & \frac{1}{N}\overset{N}{\underset{i=1}{\sum }}|v_{i}| \end{array}$$

where *v*_*i*_ is the voltage at the *i*th sampling and *N* is the number of sampling points.

**Figure 4:**
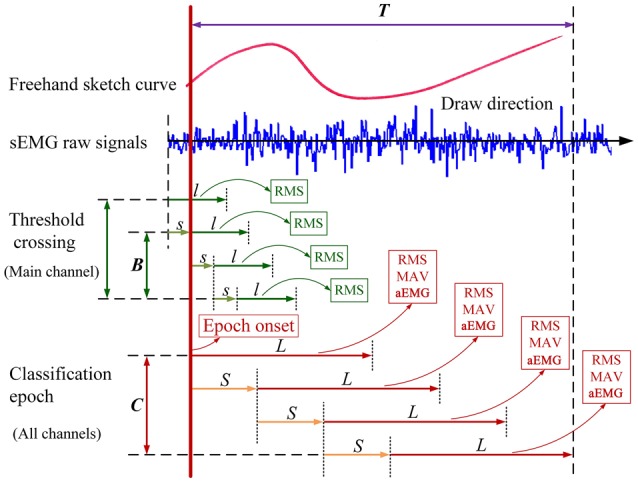
**Windowing scheme of sEMG data in the continuous feature extraction**. The size of each epoch of individual shapes is *T*. The size of sliding window for detecting threshold crossing is first set as *l* with a window increment of *s*. When the RMS value of the sliding window crossing the threshold lasts for *B* segments, the onset of the window will be the epoch onset of individual shapes. Next, the size of successive analysis windows for classification will change to *L* with a window increment of *S*. RMS, MAV, and aEMG are calculated at each *L* intervals. The sEMG data for classification is divided into *C* segments for every *L* that is the length of integrated samples as a feature extraction and the start point is shifted every *S*. Although six channels of sEMG data are used, only the main channel is shown here for illustrative purposes.

We designed a Labview script to control the feature extraction of sEMG signals. The framework is illustrated in Figure 4. There are two steps in the windowing scheme for the feature extraction of sEMG data. The first step is threshold crossing. The aim of this step is to detect the onset of individual sketching epoch for classification. The start point of the current sliding window can be decided as the epoch onset when the calculated RMS value of it is equal or greater than the presupposed threshold, and the RMS values of the following certain number of sliding windows are also crossing the threshold. The main channel that has the highest RMS value will be used for detecting threshold crossing, and the epoch onset of the main channel will be designated for all channels. The second step is feature extraction of classification epoch of individual shapes. The feature (RMS, MAV, and aEMG) values can be calculated using successive analysis windows. The size of the analysis windows here is different from that of sliding windows for detecting onset.

There are two major techniques in data windowing: adjacent windowing and overlapped windowing, depending on the difference between increment time and the segment length (Englehart and Hudgins, 2003; Oskoei and Hu, 2007). In this study, the overlapped windowing technique that results in a dense decision stream was used for detecting threshold crossing and onsets precisely, and all data were segmented for feature extraction using the adjacent windowing technique for saving computation time. The main channel in this paper is the channel collecting EMG signals from extensor digitorum (ED). In our study, sliding window for detecting threshold crossing was 20 ms (20 samples at 1000 Hz sampling), and the window increment was 5 ms. Two types of analysis window were set for comparison: short analysis window (window length of 250 ms with a 250 ms increment), and long analysis window (window length of 2500 ms with a 2500 ms increment). Rectified sEMGs from all muscles were segmented into epochs corresponding to individual shapes using a threshold that detected EMG bursts and designated the epoch onset. The threshold was set as 0.6 RMS during each set of tested shapes, which can detect all the trials (sketching epochs) per participant precisely. Then the epoch onset is determined when the RMS of the EMG signals crossing the threshold lasts for 300 ms (57 segments of sliding window for detecting threshold crossing). After these onsets had been determined, the EMG recording was segmented into 2.5 s epochs (10 segments of short analysis window; 1 segment of long analysis window) which represented the sketching of each shape. For the short analysis window, we can collect Segment (10) × Time domain characteristic (3) × Tested muscle (6), amounting to 180 parameters per participant per sketching shape. For the long analysis window, we can collect Segment (1) × Time domain feature (3) × Tested muscle (6), amounting to 18 parameters per participant per sketching shape.

After collecting all RMS, MAV, and aEMG values, to reduce data dimensionality, PCA was used to preprocess the EMG data before the classification step.

### 2.5. Classification

GEP is applied to the patterns extracted from multi-class, multi-channel continuous EMG signals for classification. Subsequently, the classifications were also obtained with two types of artificial neural networks (BPNN and ENN). The computing programs implementing the GEP were written with C++ language in Windows 7 and performed on a computer with a 2.8 G Intel Core Duo CPU and 8 G RAM. The neural network model is built with the simulation software NeuroSolutions 6 on Windows 7. The classifications were carried out with the same training and test sets.

#### 2.5.1. Gene expression programming classifier

GEP is a genetic algorithm (GA) as it uses populations of individuals, selects them according to fitness, and introduces genetic variation by using one or more genetic operators (Ferreira, 2001). As a global search technique using GA, GEP has exhibited great potential for solving complex problems (Ferreira, 2001). GEP uses fixed- length, linear strings of chromosomes to represent computer programs in the form of expression trees of different shapes and sizes, and implements a GA to find the best program (Zhou et al., 2003). One of the advantages of using GEP over other data-driven techniques is that it can produce explicit formulations of the relationship that rules the physical phenomenon (Martí et al., 2013). The reasons to use GEP for classification are the flexibility, capability, and efficiency of GEP (Zhou et al., 2003). The procedure of construction for sketching recognition was as follows.

**Figure 5:**
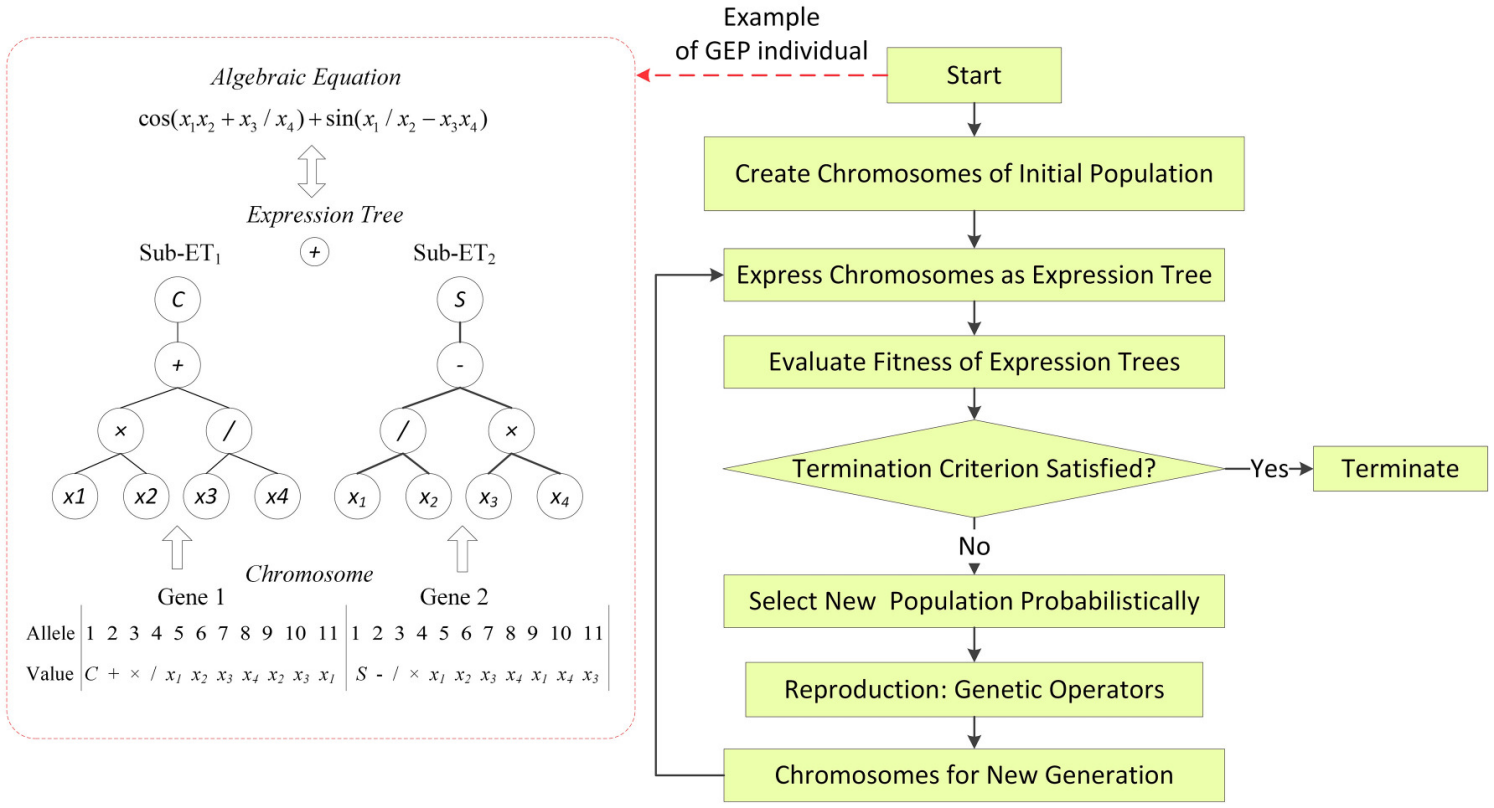
**Scheme of the GEP modeling approach (***S*** : ***sin***(), ***C*** : ***cos***())**.

Step 1: Population Initialization
Each GEP chromosome is composed of a list of symbols with a fixed length, which can be any element from the function set and the terminal set. In our study, eight elements were chosen as the mathematical function set: *F* = {+, −, ×, /, *Sin, Cos, Sqrt, Exp*}. Then, the terminal set: *T* = {*x*_1_, *x*_2_, *x*_3_, *x*_4_, *x*_5_, *x*_6_, *x*_7_, *x*_8_, *x*_9_, *x*_10_, *x*_11_, *x*_12_} was selected, if, for instance, twelve sEMG features (*x*_1_ − *x*_12_) were extracted from 6 channels. Length of head, *h* = 15, length of tail, *t* = 16, three genes per chromosomes were employed. The linked function was “addition” for algebraic sub tree for our study.
Step 2: Genetic Operation
Basic genetic operators were applied for each generation, including mutation, inversion, IS transposition, RIS transposition, one-point recombination, two-point recombination, gene recombination and gene transposition. The details about how these operators implement can be seen in Ferreira (2001).
Step 3: Fitness Calculation The maximum fitness (*f*_*max*_) was set to 1000, and then the fitness was calculated as follows:
  (4) $$\begin{array}{l} f_{fitness}=1000\times \frac{1}{MSE_{i}+1} \end{array}$$
  (5) $$\begin{array}{l} MSE_{i}=\frac{1}{m}\overset{m}{\underset{j-1}{\sum }}\left(F_{ij}-T_{j}\right) \end{array}$$
  where MSE represents the mean square error, *m* is the total number of fitness cases, *F*_*j*_ is the value output by individual program *i* for the fitness case *j* (out of *m* fitness cases) and *T*_*j*_ is the target value for the fitness case *j*. For a perfect fit, *F*_*ij*_ = *T*_*j*_.
Step 4: Termination Criterion
There were two termination criteria: (1) *f*_*fitness*_ = *f*_*max*_; (2) the maximum number of generations reached 2000. If either criterion was satisfied, stopped, else, went to step 2. The parameters used per run are summarized in Table 2. The GEP modeling approach is represented in the scheme of Figure 5.
In Figure 5, terminal *x*_1_ − *x*_4_ represent the variables; the alleles represent the position in the genes; since there is a one-to-one relationship between the symbols of the genes and the functions or terminals they represent. According to the GEP rules, the genes will be expressed as ETs and the ETs can also be easily decoded as an algebraic equation.
For a two-class (binary) problem, the GEP expression performs classification by returning a positive or nonpositive value indicating whether or not a given instance belongs to that class, i.e.,
  (6) $$\begin{array}{l} If\;GEP_{i}\left(X\right)>0,\;then\;X\;\in \;Class\;i;\;else\;X\;\notin \;Class\;i\;\left(i=0,\;1\right) \end{array}$$
where *X* is the input feature vector. For an *n*-class classification problem, where *n* > 2, one-against-all learning method is used to transform the *n*-class problem into *n* 2-class problems. These are constructed by using the examples of class *j* as the positive examples and the examples of classes other than *j* as the negative examples (Zhou et al., 2003). Our study is an 11 2-class problem.

**Table 2:** **Training parameters of GEP based models**.

**Parameter**	**Value**
Number of chromosomes	50
Function set	*F* = [+, −, ×, /, *Sin, Cos, Sqrt, Exp*]
Terminal	*T* = [*x*_1_−*x*_12_]
Number of genes, head size	3, 15
Rounding threshold	0.5
Linking function	Addition
Fitness function error type	MSE
Mutation rate	0.044
Inversion rate	0.05
IS/RIS/gene transposition rate	0.05
One-Point/ two-point recombination rate	0.2
Gene Recombination Rate	0.05

#### 2.5.2. Back propagation neural network classifier

There are many types of artificial neural networks (ANNs). The ANNs is suitable for modeling nonlinear data and is able to cover the distinction among different conditions. As one of the most common ANNs, BPNN has been widely used in pattern recognition models for sEMG signals (Nan et al., 2003). Back propagation (BP) learning comprises two processing steps involving the forward and BP of error. The BP architecture is the most popular model for complex, multi-layered networks (Xing et al., 2015). A three-layer network consisting of one input layer, one hidden layer with a sigmoid function, and one output layer with a tanh function was used to set up the BPNN classification models.

#### 2.5.3. Elman neural network classifier

Compared to the BPNN, the ENN was not frequently used for classifying sEMG signals of motion patterns. However, in our early work in Chen et al. (2015), the recognition rate of the ENN-based model was slightly superior to the performance of the BPNN-based model for eyebrow emotional expression recognition. As a subclass of recurrent neural networks, the ENN has a short-term memory function, which has been found to be particularly useful for the prediction of discrete time series, owing to its abilities to model nonlinear dynamic systems and to learn time-varying patterns (Ardalani-Farsa and Zolfaghari, 2010). To set up the ENN model, we used a four-layer network consisting of one input layer, one hidden layer with a sigmoid function, one context layer, and one output layer with a tanh function. More detailed description of the structure of ENN can be found in Chen et al. (2015).

#### 2.5.4. Performance evaluation criteria

In this paper, the accuracy rate (*AR*) and *AR*_11_ were used to evaluate the classification performance of five classifiers mentioned above. The *AR* can be calculated as follows:

(7) $$\begin{array}{l} AR=\frac{c}{C}\times 100\% \end{array}$$

Where *c* is the number of correctly classified test samples, and *C* is the total number of tested samples. A larger *AR* value (close to one) indicate that the performance of the classifier is better. *AR*_11_ was the mean *AR* of 11 sketching shapes for each subject.

## 3. Results and discussion

Feature extraction and recognition algorithms had to be performed on the data from individual subjects and did not generalize to other subjects because of inter-subject variability (Linderman et al., 2009). The dataset was randomly divided into two subsets, a training set, and test set, for recognition. 70% of the records from each day were used as the training set, and the remainder of the records was used as the test set. The training set contained 770 sEMG samples, whereas the test set contained 330 samples. For our study, the normalization interval was set as [0.05, 0.95].

### 3.1. Results of feature extraction

**Figure 6:**
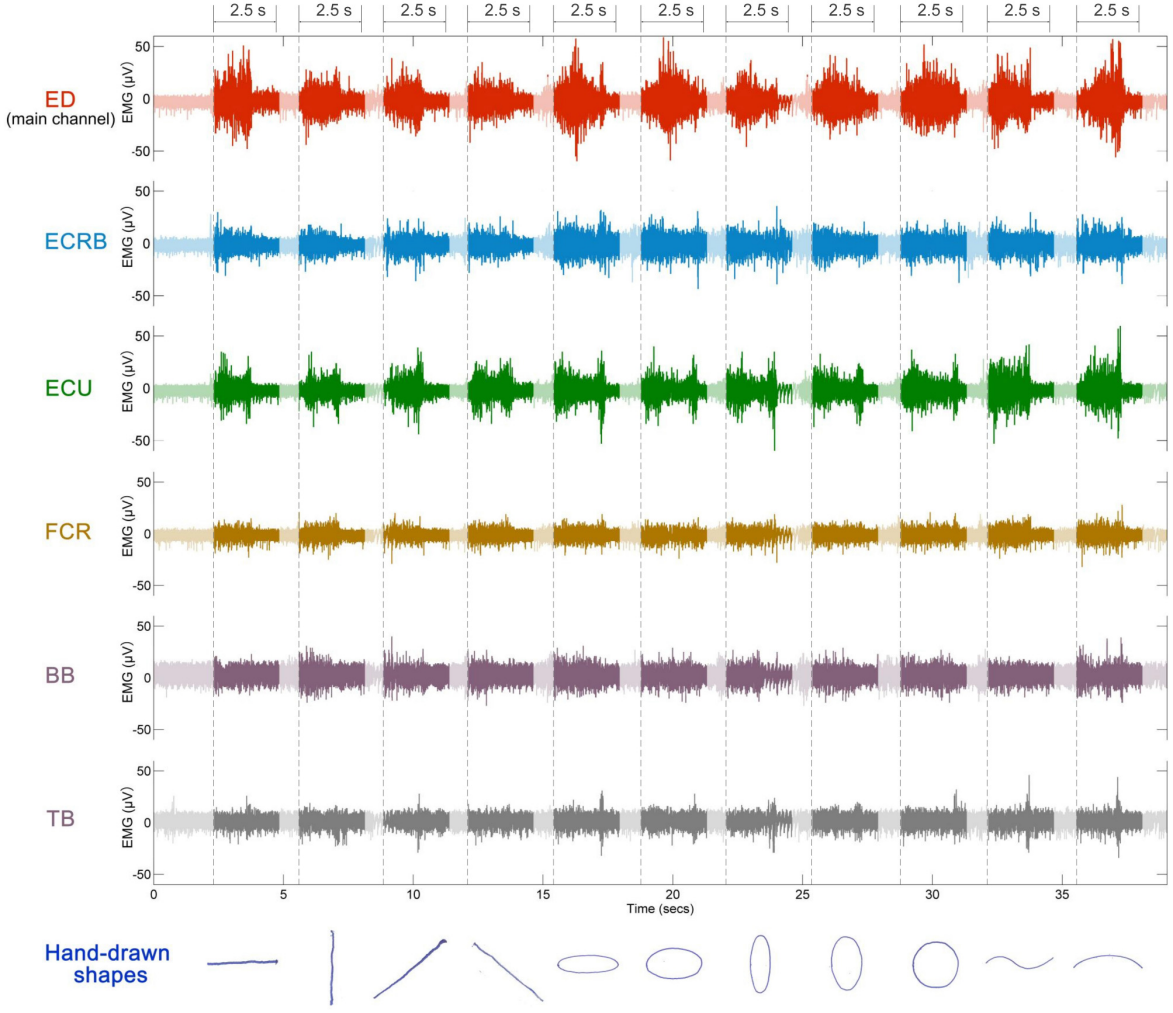
**Example of discrimination for a representative recording session**. Six sEMG channels were used for sketching recognition. Dotted lines represent epoch onsets. Segments (2.5 s) are marked on the top.

Representative examples of the EMG signals and sketching traces are shown in Figure 6. The channel of ED that had the highest RMS value among the six sEMG channels was used as the main channel for epoch onset discrimination. Setting the threshold as 0.6 RMS can help detect 1100 trials per participant precisely. After these onsets had been determined, the EMG record was segmented into 2.5 s epochs which represented the sketching duration of each shape.

For the short analysis window, the number of parameters was reduced from 180 to 12 principal components using PCA. For the long analysis window, the number of parameters was reduced from 18 to 5 principal components.

### 3.2. Classification results

**Figure 7:**
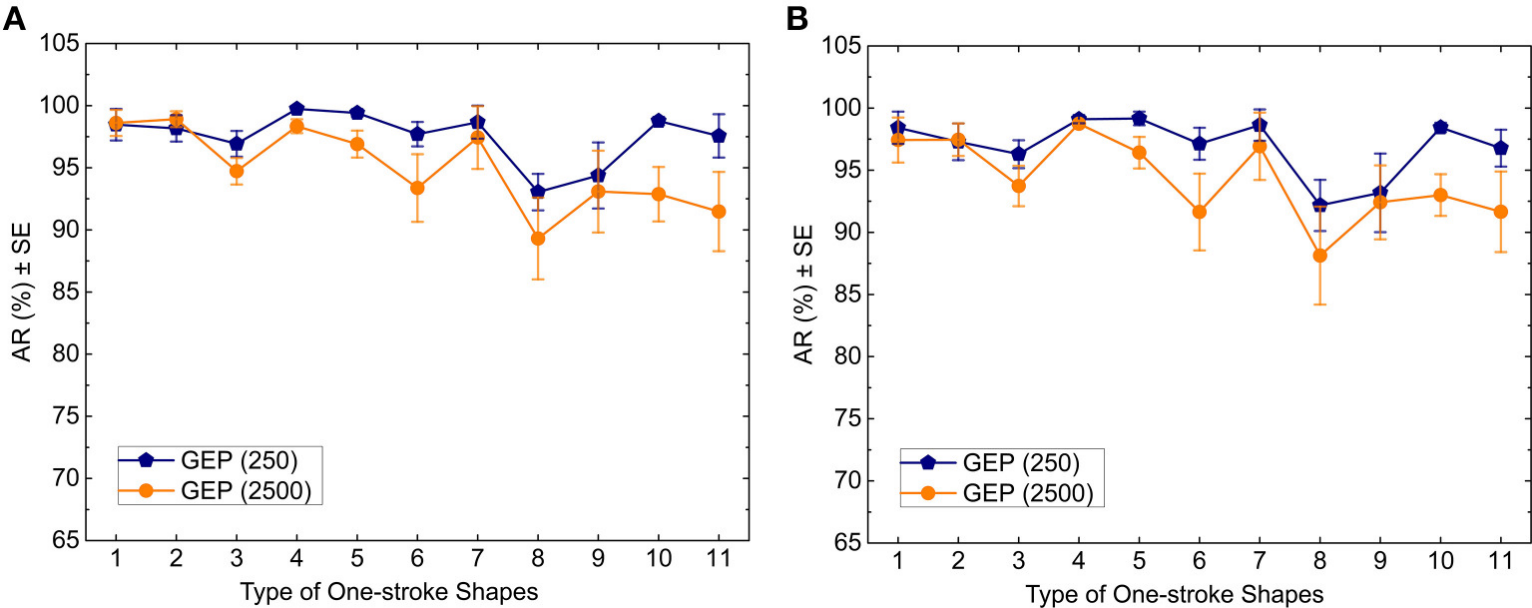
**Classification ***AR*** achieved by the GEP model vs. type of one-stroke shapes for 250 ms analysis windows and 2500 ms analysis windows as measured with (A) the training data and (B) the test data**.

The 12 principal components extracted using short analysis windows (250 ms) and principal components extracted using long analysis windows (2500 ms) were entered as a 12-element feature vector and a 5-element feature vector into the GEP classifiers respectively. The averages of *AR* of the 11 one-stroke shapes are shown in Figure 7. These averages are the means of AR for the four participants. The error bars represent the standard error (SE).

According to Figure 7, for the training set, using the short analysis window, the average values of *AR* from left to right are 98.47, 98.17, 96.93, 99.74, 99.41, 97.7, 98.67, 93.04, 94.38, 98.76, and 97.56%; using the long analysis window, the average values of *AR* from left to right are 98.61, 98.91, 94.72, 98.34, 96.91, 93.37, 97.43, 89.31, 93.08, 92.88, and 91.48%. For the test set, using the short analysis window, the average values of *AR* from left to right are 98.42, 97.46, 96.29, 99.11, 99.17, 97.13, 98.64, 92.17, 93.18, 98.45, and 96.78%; using the long analysis window, the average values of *AR* from left to right are 97.43, 97.28, 93.73, 98.75, 96.42, 91.64, 96.93, 88.14, 92.42, 93, and 91.66%.

**Figure 8:**
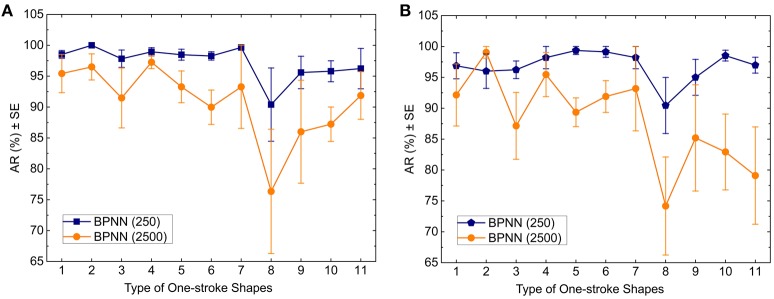
**Classification ***AR*** achieved by the BPNN model vs. type of one-stroke shapes for 250 ms analysis windows and 2500 ms analysis windows as measured with (A) the training data and (B) the test data**.

For comparison purpose, we used the same training and test set for the BPNN classifier. For the short analysis window, we set the number of hidden layer to 1 and the number of hidden layer nodes to 25. For the long analysis window, we set the number of hidden layer to 1 and the number of hidden layer nodes to 11. We defined two termination criteria for the training phase: the maximum number of iterations was 2000, and the minimum mean square error (MSE) was less than 0.01. If either criterion were satisfied, the algorithms would stop. The averages of *AR* of the 11 one-stroke shapes are shown in Figure 8.

According to Figure 8, for the training set, using the short analysis window, the average values of *AR* from left to right are 98.53, 100, 97.83, 98.95, 98.48, 98.28, 99.65, 90.4, 95.6, 95.8, and 96.23%; using the long analysis window, the average values of *AR* from left to right are 95.43, 96.5, 91.48, 97.25, 93.28, 89.98, 93.28, 76.35, 86, 87.23, and 91.88%. For the test set, using the short analysis window, the average values of *AR* from left to right are 96.88, 96, 96.23, 98.2, 99.35, 99.12, 98.2, 90.45, 95, 98.53, and 96.97%; using the long analysis window, the average values of *AR* from left to right are 92.15, 99.05, 87.15, 95.45, 89.35, 91.9, 93.17, 74.18, 85.2, 82.93, and 79.1%.

**Figure 9:**
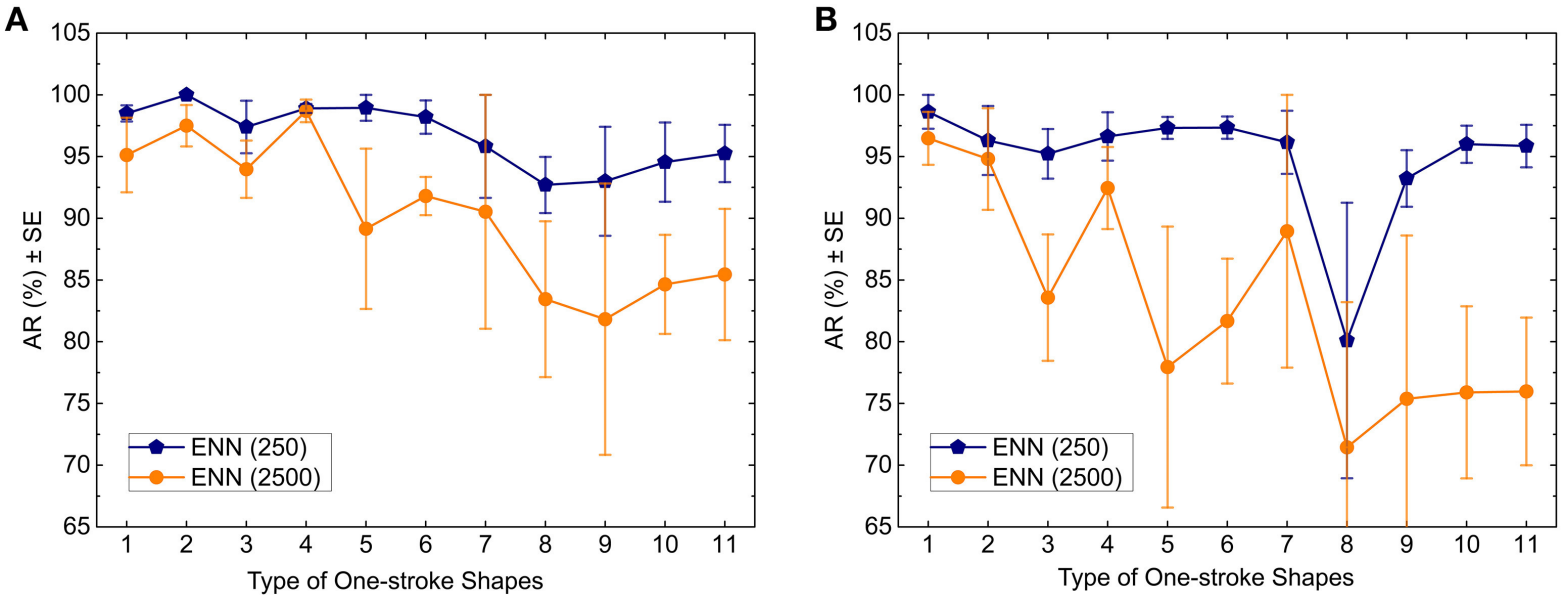
**Classification ***AR*** achieved by the ENN model vs. type of one-stroke shapes for 250 ms analysis windows and 2500 ms analysis windows as measured with (A) the training data and (B) the test data**.

For comparison purpose, we also used the same training and test set for the ENN classifier. We establish the same number of hidden layer, the number of hidden layer nodes and termination criteria as the BPNN. The averages of *AR* of the 11 one-stroke shapes are shown in Figure 9.

According to Figure 9, for the training set, using the short analysis window, the average values of *AR* from left to right are 98.5, 100, 97.4, 98.9, 98.95, 98.2, 95.83, 92.7, 93, 94.55, and 95.25%; using the long analysis window, the average values of *AR* from left to right are 95.13, 97.5, 93.98, 98.7, 89.15, 91.8, 90.53, 83.45, 81.83, 84.65, and 85.45%. For the test set, using the short analysis window, the average values of *AR* from left to right are 98.63, 96.3, 95.23, 96.63, 97.33, 97.35, 96.15, 80.1, 93.23, 96, and 95.85%; using the long analysis window, the average values of *AR* from left to right are 96.48, 94.8, 83.58, 92.45, 77.95, 81.68, 88.95, 71.45, 75.38, 75.9, and 75.98%.

**Figure 10:**
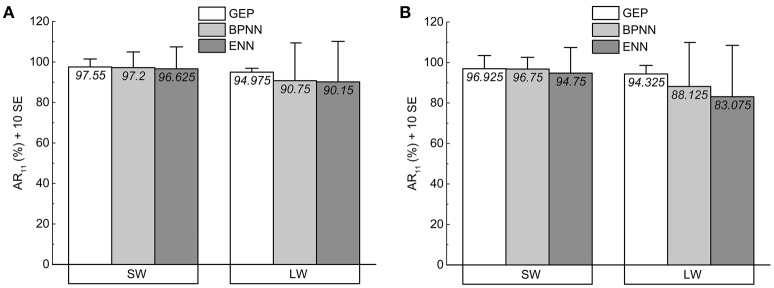
**Histogram bars and error bars of ***AR***_**11**_ as measured with (A) the training data and (B) the test data**. (Error bars denoteSE of the means; SW, short analysis window; LW, long analysis window).

Tables 3, 4 show the *AR*_11_ for individual subjects achieved by the three recognition models. The averages of *AR*_11_ achieved by the three recognition models are shown in Figure 10. These averages are the means of *AR*_11_ for the four participants. The error bars represent the standard error (SE).

**Table 3:** *****AR***_**11**_ for individual subjects for the training set**.

**Subjects**	**Analysis window**	**GEP**	**BPNN**	**ENN**
	**(ms)**	***AR*_11_ (%)**	***AR*_11_ (%)**	***AR*_11_ (%)**
Man 1	250	98.1	97.2	93.6
	2500	95.1	89.1	85.4
Man 2	250	97.7	97.7	98.1
	2500	95.2	96.2	95.1
Woman 1	250	96.4	95.1	96.5
	2500	94.4	87.8	90.8
Woman 2	250	98.0	98.8	98.3
	2500	95.2	89.9	89.3

**Table 4:** *****AR***_**11**_ for individual subjects for the test set**.

**Subjects**	**Analysis window**	**GEP**	**BPNN**	**ENN**
	**(ms)**	***AR*_11_ (%)**	***AR*_11_ (%)**	***AR*_11_ (%)**
Man 1	250	97.9	96.5	94.1
	2500	94.9	85.9	79.4
Man 2	250	97.4	98.3	97.9
	2500	94.9	94.6	90.6
Woman 1	250	95.0	96.7	91.8
	2500	93.1	86.7	81.2
Woman 2	250	97.4	95.5	95.2
	2500	94.4	85.3	81.1

We can conclude that compared with the BPNN and ENN classifiers, the GEP classifier performed best for the training data and generalized well to the test data: for both data sets and lengths of analysis window, all the values of *AR*_11_ achieved by the GEP model are greater than 93%; the averages of *AR* of 11 one-stroke shapes achieved by the GEP model are higher than those of BPNN and ENN.

It can also be observed that compared with the long analysis window, the short analysis window can help improve the *AR* by around 6.4% averagely for all three classifiers. However, for GEP classifier, using short analysis window can only improve the *AR* by around 2.6% averagely, significantly less than those achieved by the BPNN and the ENN classifiers (7.6%, 9.1%), which can also indicate that the GEP classifier is able to perform well with either length of analysis window.

### 3.3. Discussion

Thus, we have shown that EMGs of arm muscles can be converted into sketching patterns. This technique potentially can substitute for current computer input devices or touch screens for sketching transmission. For example, it can provide another method for implementing sketching in the air. In recent years, computer vision technology can help recognize handwritten characters and sketches in the air (Chen et al., 2008; Asano and Honda, 2010; Hammond and Paulson, 2011; Vikram et al., 2013). However, it appears that attaining a high recognition rate using computer-vision based methods is currently only possible with high-quality input images or videos and is vulnerable to factors such as camera angle, background and lighting (Zhao, 2012; Chen et al., 2015). This disadvantage of the computer-vision based methods is avoided by the sEMG-based method. The sEMG-based approaches have been successfully used for the recognition of handwriting (Linderman et al., 2009). However, to our knowledge, there were rare previous studies using sEMG to recognize sketching.

Each 2.5 s sketching epoch was detected using 0.6 RMS as the threshold for feature extraction, and then dimensions were reduced with PCA. The GEP classifier was able to recognize 11 one-stroke shapes with nearly perfect accuracy using 12 or 5 principal components of sEMG signals as input vectors. Very limited number of studies has been done on using this approach to extract sEMG features for sketching recognition; however, this novel approach of feature extraction (Figure 4) reveals the basis of the excellent classification result of the GEP classifier.

Some researchers used sliding and analysis windows for feature extraction of sEMG signals in the field of real-time myoelectric control of prostheses and exoskeletons (Englehart and Hudgins, 2003; Oskoei and Hu, 2007; Geethanjali and Ray, 2011; Tang et al., 2014). We compared two different lengths of adjacent analysis window, and the results indicate that the short analysis window can get higher accuracy rates than the long one (Figures 7–9, Tables 3, 4), which is in accordance with the finding of Englehart and Hudgins (2003) who proposed that a smaller segment increment produces a denser but semi-redundant stream of class decisions that could improve response time and accuracy. The short analysis window can divide one-stroke sketching process into more segments, from which more important details of features can be extracted. A larger amount of data will result in features with lower statistical variance and, therefore, greater classification accuracy. The length of the short analysis window (250 ms) also conforms with the optimal window length (150–250 ms) proposed by Smith et al. (2011) for pattern recognition-based myoelectric control.

Interestingly, it can be observed from Figures 7–9 that the vertical 40° ellipse was recognized worst for each classifier. The reason may be that the vertical 40° ellipse is similar to two shapes (the vertical 20° ellipse and the perfect circle), which can lead to the increasing rate of false recognitions of it.

It can also be found that the proposed GEP classifier presented the highest accuracy and robust when compared with the BPNN classifier and the ENN classifier. One of the advantages of the GEP classifier is that it can produce simple explicit formulations (Landeras et al., 2012; Yang et al., 2016), which gives a better understanding of the derived relationship between the sEMG signals and one-stroke sketching shapes and makes the model suitable for application in real time. Although the ENN classifier achieved higher accuracy rate than the BPNN classifier in our previous work (Chen et al., 2015), it performed worst in this study. The recognition results (*AR*, *AR*_11_) of the GEP classifier were slightly better than those of the BPNN and ENN classifiers with the short analysis window. This finding suggests that the sEMG-based sketching recognition method with the short analysis window should be robust to variations of the recognition algorithm.

Overall, our method may contribute to an efficient and natural way to sketch freely and precisely in computers or digital devices, and may be appropriate for clinical applications, including computer-aided design, virtual reality, prosthetics, remote control, entertainment as well as muscle-computer interfaces in general. For further optimization of our method, we plan to deal with the contradictions between the accuracy of recognition and natural applications through finding the optimal combination of muscle channels, window lengths, and sEMG features. In future work, we plan to develop a new HCI tool with a wearable armband that can be used as a muscle-computer interface (Chowdhury et al., 2013) for sketching in the air. However, our findings and the general approach have several limitations:

1. Three time domain indices of sEMG signals were extracted as features. To further improve the robustness and discriminatory accuracy of similar shapes (e.g., vertical 20° ellipse, perfect circle and vertical 40° ellipse), other time domain indices, frequency domain indices and time-frequency domain indices could be additionally extracted.
2. In our study, subjects were required to sketch on a template paper with fixed dimensions (Figure 1). However, these basic shapes can also be shown with different degrees and scales in practical drawings. Whether our method can recognize these additional one-stroke shapes needs further research.
3. We specified the sequence of shapes, the starting point and the direction of movements using sketching templates. This makes the problem simplified as compared to a real-life scenario, in which people have their habits of drawing the same shape. To prove the actual usability of the method, our future work will consider more flexible and variable ways of freehand sketching in a more general setting.
4. To test our approach, we selected 11 one-stroke shapes, which are variations of four basic one-stroke shapes (Robertson and Bertling, 2013) and frequently used among the users in product design. However, there is no definitive set of basic sketching shapes. In the future work, we will enlarge the number of the tested shapes and offer more general basic shapes that users would need or not, in the intended context.
5. Our method recognized 11 one-stroke shapes from the EMG patterns, showing outstanding classification rates on discrete symbols and shapes. Thus, we can note that discrete symbol classification has been a relatively easy task. Recently, a much more challenging task is continuous decoding of handwritten/drawn traces. This was attempted by Linderman et al. (2009), Huang et al. (2010), and Li et al. (2013) and improved recently by Okorokova et al. (2015). The later one attempted to use dynamical properties of the written coordinates to aid continuous decoding of the coordinates based on EMG. Another attempt of using more information was done by Rupasov et al. (2012), who complemented EMG during handwriting with EEG recordings, but they only found weak correlations between the two sets of data. One goal of our future work is continuous sketching recognition or reconstruction. Therefore, we will try to construct the prediction model between EMG signals and the coordinates (*x, y*) of pen traces for reconstruction of free-form curves using some linear or nonlinear decoding algorithms.
6. Although it seems that there was sufficient data to achieve extremely high accuracy with our method, the recognition performance can be further improved with more training samples. Our method is heavily dependent on the size of the training dataset. In future work, we can use some other state-of-the-art methods that can achieve high performance with small training samples, which is convenient for users.

## 4. Conclusion

In this paper, we attempted to verify whether a robust variant of GP, namely GEP could be derived to recognize sketching patterns from arm sEMG signals. While the results are encouraging, additional research is needed to develop the method further. The technique mentioned in this work potentially can bring significant change for the conventional human-machine interface, and make great convenience for the healthy persons and the disabled with hand deficiency. Our future work will concentrate on the development of a wearable armband that can be used as a natural perceptual interface for sketching in the air.

## Author contributions

Substantial contributions to the conception or design of the work; or the acquisition, analysis, or interpretation (ZY, YC). Drafting the work or revising it critically for important intellectual content (ZY, YC). Final approval of the version to be published (ZY, YC). Agreement to be accountable for all aspects of the work in ensuring that questions related to the accuracy or integrity of any part of the work are appropriately investigated and resolved (ZY, YC).

### Conflict of interest statement

The authors declare that the research was conducted in the absence of any commercial or financial relationships that could be construed as a potential conflict of interest.
